# Root transcriptional responses of two melon genotypes with contrasting resistance to *Monosporascus cannonballus* (Pollack *et* Uecker) infection

**DOI:** 10.1186/1471-2164-13-601

**Published:** 2012-11-08

**Authors:** Cristina Roig, Ana Fita, Gabino Ríos, John P Hammond, Fernando Nuez, Belén Picó

**Affiliations:** 1 Institute for the Conservation and Breeding of the Agricultural Biodiversity, Universitat Politècnica de València (COMAV-UPV), Camino de Vera s/n, 46022, Valencia, Spain; 2 Instituto Valenciano de Investigaciones Agrarias (IVIA), Carretera Moncada-Náquera km 4.5, 46113, Moncada, Valencia, Spain; 3 School of Plant Biology, University of Western Australia, 35 Stirling Highway, Crawley, 6009, WA, Australia

## Abstract

**Background:**

*Monosporascus cannonballus* is the main causal agent of melon vine decline disease. Several studies have been carried out mainly focused on the study of the penetration of this pathogen into melon roots, the evaluation of symptoms severity on infected roots, and screening assays for breeding programs. However, a detailed molecular view on the early interaction between *M. cannonballus* and melon roots in either susceptible or resistant genotypes is lacking. In the present study, we used a melon oligo-based microarray to investigate the gene expression responses of two melon genotypes, *Cucumis melo* ‘Piel de sapo’ (‘PS’) and *C. melo* ‘Pat 81’, with contrasting resistance to the disease. This study was carried out at 1 and 3 days after infection (DPI) by *M. cannonballus*.

**Results:**

Our results indicate a dissimilar behavior of the susceptible *vs.* the resistant genotypes from 1 to 3 DPI. ‘PS’ responded with a more rapid infection response than ‘Pat 81’ at 1 DPI. At 3 DPI the total number of differentially expressed genes identified in ‘PS’ declined from 451 to 359, while the total number of differentially expressed transcripts in ‘Pat 81’ increased from 187 to 849. Several deregulated transcripts coded for components of Ca^2+^ and jasmonic acid (JA) signalling pathways, as well as for other proteins related to defence mechanisms. Transcriptional differences in the activation of the JA-mediated response in ‘Pat 81’ compared to ‘PS’ suggested that JA response might be partially responsible for their observed differences in resistance.

**Conclusions:**

As a result of this study we have identified for the first time a set of candidate genes involved in the root response to the infection of the pathogen causing melon vine decline. This information is useful for understanding the disease progression and resistance mechanisms few days after inoculation.

## Background

*Monosporascus cannonballus* (Pollack *et* Uecker) is an ascomycete soil-borne pathogen adapted to hot, arid and semi-arid areas that causes root/rot vine decline. This disease has severe economic impact on global melon (*Cucumis melo* L) and watermelon (*Citrullus lanatus* (Thunb.) Matsum. & Nakai) production [1]. Infection of roots may occur *via* germinating ascospores and/or active mycelium in infested soils. When roots are infected, they become necrotic with numerous discrete lesions causing the loss of most of secondary and tertiary roots. During the growing season, symptoms are characterized by reduced plant growth, progressive defoliation and partial or complete canopy collapse, resulting in fruit sunburn and total crop loss at harvest.

Ascospores probably function as the primary survival structure, as well as the primary inoculum for root infection [2,3]. Studies on the specificity of *M. cannonballus* showed that germination of ascospores is extremely host specific and occurs only in the rhizosphere of certain genera and species of plants belonging exclusively to the Cucurbitaceae family [4]. In a histological study using artificial inoculation with active mycelium, Alfaro-Fernandez and García-Luis [5] examined the early colonization of *M. cannonballus* in two cucurbit species that differed in their sensitivity to this disease: a highly sensitive muskmelon (*C. melo*) and a tolerant squash (*Cucurbita maxima*). Results showed that *M. cannonballus* was capable of infecting the tissue of both host plants, colonizing the epidermis and cortex with decreased density of mycelium at the endodermis level. Differences in sensitivity of muskmelon and squash seemed to be due to the differential resistance to the initial penetration of the fungus.

Studies show that *M. cannonballus* is a facultative saprophyte fungus, does not form special penetration structures such as appressoria and pathogen reproduction in infected roots occurs primarily after plant death [6]. It behaves in a manner most similar to that of vascular wilt pathogens, but differs in that it is not systemic and cannot be isolated from aerial portions of infected plants [7].

Strategies for controlling vine decline based on the development of resistant cultivars have been initiated in most affected countries. Since large genetic variability is displayed within *C. melo* varieties, the selection of resistant material has become a major objective for plant breeding approaches [8]. *C. melo* germplasm is commonly classified into two subspecies, ssp. *melo* including the main commercial types, and ssp. *agrestis* with important sources of resistance and quality traits [9]. The genotype ‘Pat 81’ of *C. melo ssp. agrestis* showed resistance to *M. cannonballus* under field conditions [10,11], which was employed to initiate a breeding program aimed at introducing resistance into Spanish melon cultivars such as *C. melo* ssp. *melo* ‘Piel de sapo’ (‘PS’) [12-14]. The resistance to vine decline of ‘Pat 81’ was expressed as a delay in the appearance of root lesions and as a slow rate of disease development with a low percentage of wilted plants at the end of the growing cycle. Root lesions caused by *M. cannonballus* infection in ‘Pat 81’ were less severe than in susceptible genotypes, being limited to small damaged areas without loss of root biomass. A vigorous and branched root structure with high regeneration potential could also help to maintain a good hydraulic conductivity and improve resistance to the infection towards the end of the season [15].

During the last decade, relevant genetic and genomic tools have been developed in melon. Available resources include new mapping populations [16,17], genetic maps [18-21], BAC-based physical maps [22], melon transcriptome characterization [23], TILLING and ECOTILLING platforms [24-26] and large EST collections [27,28]. Recently, an oligonucleotide-based microarray has been developed from a dataset of 17,510 unigenes obtained from normalized cDNA libraries, representing different melon genotypes, tissues and physiological conditions, including *M. cannonballus* infected root tissue [29]. A preliminary comparison between ‘Pat 81’ infected *vs.* non-infected roots was used for the validation of this microarray platform [29]. Inoculation was carried out on artificially infected soil and samples were collected 14 days after inoculation. At this late stage of the infection, only a low number of genes were found to be differentially regulated between *M. cannonballus* infected and non-infected roots of ‘Pat 81’.

To date, a detailed molecular view on the early interaction between *M. cannonballus* and melon roots in either susceptible or resistant genotypes is lacking. To improve our knowledge on the genetic regulation of root/rot vine decline resistance in melon, the transcriptional changes associated with *M. cannonballus* inoculation of the susceptible ‘PS’ *vs.* the resistant ‘Pat 81’ are studied here at two time points of early infection.

## Results and discussion

### Root infection with *M. cannonballus* occurs rapidly in ‘PS’

The study of the early response of melon roots to fungal infection by *M. cannonballus* required a method for rapid infection of roots different from the traditional soil inoculation procedures employed previously [30]. A method based on the direct contact of melon roots with *M. cannonballus* mycelium, and subsequent growth in a hydroponic system optimally met these requirements.

**Figure 1:**
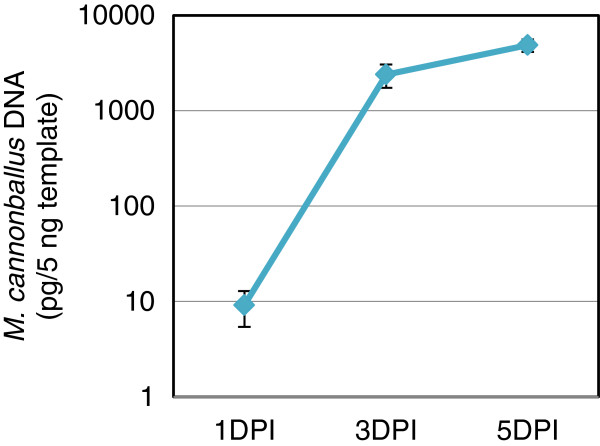
**Quantification of *M. cannonballus *infection in roots of ‘Piel de sapo’ (‘PS’) plants by quantitative real-time PCR.** The amount of a *M. cannonballus* target DNA, measured as pg per 5 ng of total genomic DNA, was used to estimate the extent of the root colonization by the fungus. Infected roots were sampled at 1, 3 and 5 days post-inoculation (DPI)

Infected roots of ‘PS’ and their respective mock-inoculated controls were collected at 1, 3 and 5 days post inoculation (DPI). There were no visible symptoms of disease at these time points; therefore, quantitative PCR was used to detect the pathogen in these roots [31]. The amount of *M. cannonballus* DNA detected in root samples increased significantly from 1 to 3 DPI (2 to 500 pg of *M. cannonballus* DNA per ng of total DNA), and subsequently remained constant until 5 DPI (Figure 1). Consequently, samples from 1 and 3 DPI were chosen for transcriptional profiling of the early stages of *M. cannonballus* infection in the roots of the susceptible genotype ‘PS’ and the resistant genotype ‘Pat 81’.

### Global gene expression trends reflect susceptibility to *M. cannonballus* root infection

Changes in the expression of genes during *M. cannonballus* root infection were identified using a melon oligonucleotide microarray (Roche NimbleGen, Madison, WI, USA). This microarray was designed using 17,443 unigenes assembled from different melon cDNA libraries, including two libraries obtained from roots infected with *M. cannonballus*[27,29]. Inoculated and mock-inoculated roots of the susceptible ‘PS’ and the resistant ‘Pat 81’ were collected at 1 DPI and 3 DPI. Three biological replicates of cDNA obtained for each condition were labelled and used for microarray hybridization.

**Figure 2:**
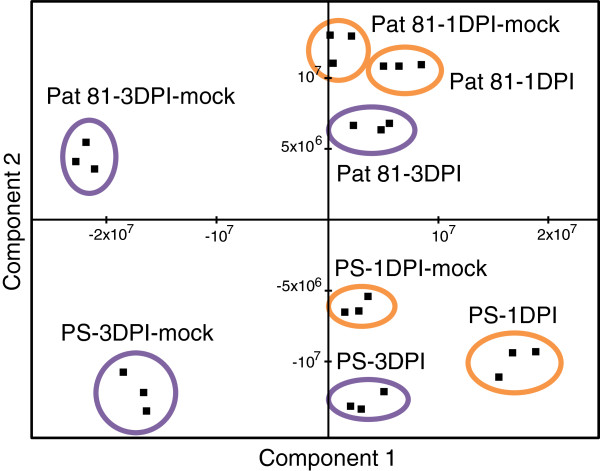
**Principal component analysis (PCA) plot of global transcriptomic results.** ‘PS’ and ‘Pat 81’ genotypes inoculated and mock-inoculated at 1 DPI and 3 DPI. The three biological replicates of each sample are plotted separately and encircled by an orange (1 DPI) or violet (3 DPI) line

Principal component analysis (PCA) was used for preliminary data analysis and quality control. The first and second components explained 29.7% and 20.7% of total variance, respectively. Biological replicates from each accession and stage (inoculated and mock-inoculated) clustered together in all cases (Figure 2).

Both root developmental stage and fungal infection seem to be associated with the first component (Figure 2). The differences observed between roots collected 1 day and 3 days in control plants, similar in both genotypes, suggest that there exist a large number of genes deregulated during root development. We also observed striking differences between inoculated and mock-inoculated roots (both at 1 and 3 DPI), with differential patterns in ‘PS’ and ‘Pat 81’. In fact, at 3 DPI, the infection produced similar plot shifts in both genotypes. However, the global gene expression differences between 1 DPI-infected and mock sample in ‘Pat 81’ background were lower when compared to the equivalent ’PS’ samples (Figure 2). This observation is consistent with slower *M. cannonballus* infection of ‘Pat 81’. The second component explained genotypic differences between ‘PS’and ‘Pat 81’ samples, suggesting the influence of genotype on hybridization signals.

**Figure 3:**
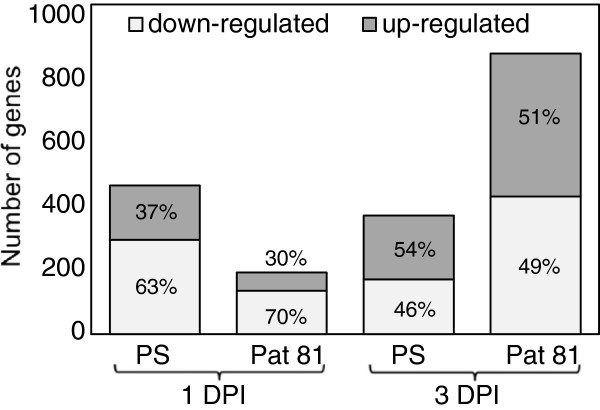
**Percentage of transcripts differentially expressed in ‘PS’ and ‘Pat 81’ after *M. cannonballus *inoculation.** Number of genes induced (dark gray bars) or repressed (light gray bars) in ‘PS’ and ‘Pat 81’ at 1DPI and 3 DPI

At 1 DPI, the abundance of 165 transcripts significantly increased and 286 decreased in ‘PS’, whereas the abundance of 56 transcripts increased and 131 decreased in ‘Pat 81’ (Additional file 1 and Additional file 2). Thus, most of modulated transcripts at 1 DPI reduced their abundance following *M. cannonballus* infection (Figure 3). A similar enrichment in down-regulated genes was observed during the susceptible plant-pathogen interaction of cotton roots with soil-borne, vascular wilt fungus *Fusarium oxysporum* f. sp. *vasinfectum*, particularly at early stages of infection [32]. However, at 3 DPI, the ratio of induced/repressed transcripts was close to one and similar in both genotypes (Figure 3; Additional file 3 and Additional file 4). The total number of differentially expressed genes identified in ‘PS’ declined from 451 (1 DPI) to 359 (3 DPI), while the total number of differentially expressed transcripts in ‘Pat 81’ increased from 187 (1 DPI) to 849 (3 DPI), which emphasizes the dissimilar behaviour of the susceptible and the resistant genotypes from the initial stages of infection.

**Figure 4:**
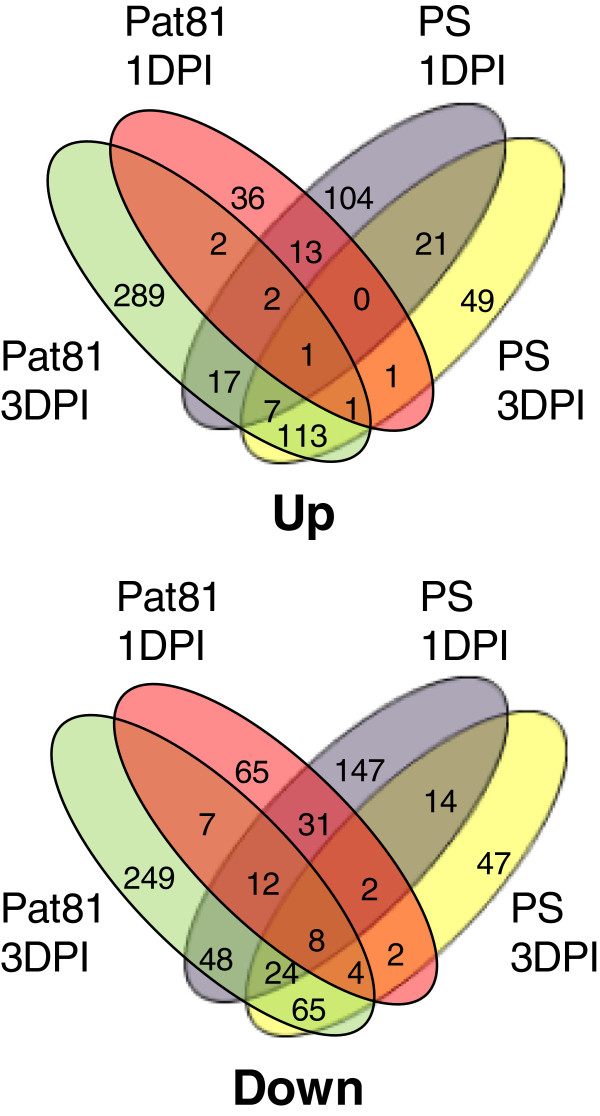
**Venn diagram of differentially expressed genes.** Genes up-regulated (upper panel) and down-regulated (lower panel) at least 2-fold in ‘PS’ and ‘Pat 81’ at 1DPI and 3 DPI

The overlap of differentially expressed transcripts shared by two or more pairs of genotype-time groups, was small for most combinations (Figure 4). The percentage of total (induced and repressed) unique transcripts in ‘Pat 81’-1DPI, ‘Pat 81’-3DPI, ‘PS’-1DPI and ‘PS’-3DPI were 54%, 63%, 56% and 27% respectively. Transcriptional profiles for ‘PS’ at 3 DPI and ‘Pat 81’ at 3 DPI had a high number of common induced (113) and repressed transcripts (65), suggesting that at 3 DPI both susceptible and resistant genotypes, had initiated a set of common transcriptional responses to *M. cannonballus* infection. In contrast, the number of common transcripts induced (13) or repressed (31) at 1DPI between both genotypes was smaller (Figure 4), consistently with the differential response of these two genotypes at the very early stages of infection.

When comparing these data with previous results studying the transcriptomic response of melon to *M. cannonballus*[29] we found only 11 coincident genes, 4 of them repressed and 7 induced (Additional file 5). None of these genes was directly related to known plant defence mechanisms. Such divergence between experiments could be due to some differences in infection time (14 days in [29] versus 1–3 days in this work) and the inoculation procedure. Whereas in the previous work the plants were grown on soil infected with 50 colony-forming units of *M. cannonballus* per gram, in this work roots were inoculated by direct contact with *M. cannonballus* mycelium and plants were grown on liquid medium, allowing a faster and more uniform infection.

### Quantitative reverse transcription-PCR validation of microarray data

**Figure 5:**
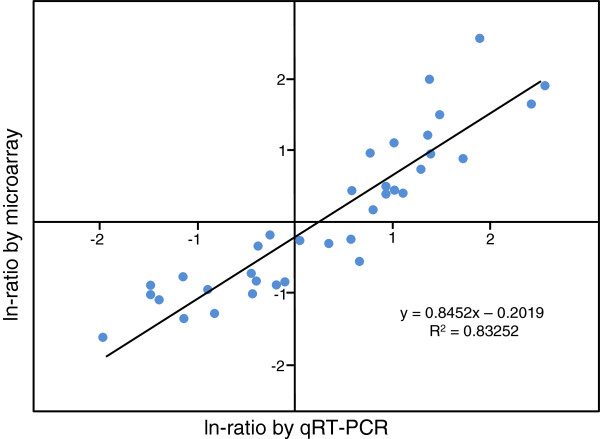
**Correlation between array and qRT-PCR data.** Fold differences for both sets of data were transformed using the natural log (ln)

In order to confirm microarray results, nine differentially expressed transcripts with different expression profiles were selected and tested by quantitative reverse transcription-PCR (qRT-PCR) analysis in RNA samples from the four assayed genotype/ time combinations (primers in Additional file 6). Selected genes were mainly involved in defence (induced and repressed in different conditions), signal transduction, metabolism, and stress response. All transcript profiles corresponding to differentially expressed genes in each condition showed similar magnitudes and directions of change, with a correlation coefficient of 0.83 (Figure 5). These results confirm the reliability of our microarray data.

### Functional analysis

**Figure 6:**
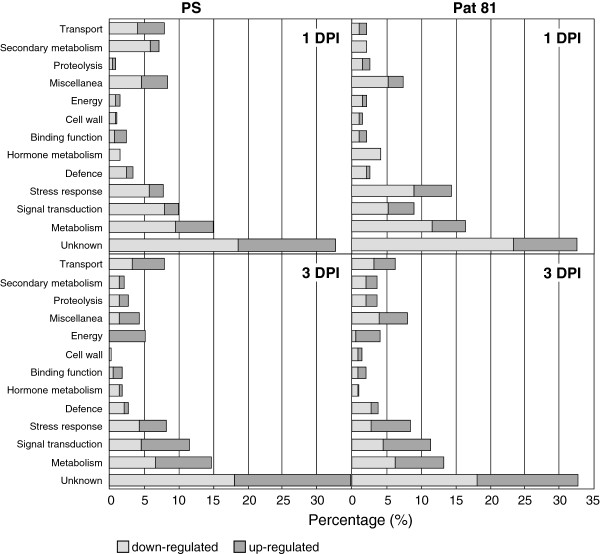
**Functional categories of differentially expressed transcripts.** Transcripts differentially expressed in ‘PS’ (left plots), and ‘Pat 81’ (right plots), at 1DPI (upper plots) and 3 DPI (lower plots) are grouped in different functional classes. Bars indicate the percentage of each single functional category within each combination of genotype and time. Induced genes are represented by dark gray bars and repressed genes by light gray bars

Differentially expressed transcripts were assigned to functional categories using MELOGEN database information [27], published data, and functional annotation performed with the BLAST2go package [33]. The percentage of transcripts assigned to each functional category in each treatment is summarized in Figure 6. “Signal transduction” category represented 9% of total genes in ‘PS’ and 10% in ‘Pat 81’ at 1 DPI, with only one common gene. At 3 DPI, the ratio of differentially expressed genes in this category slightly increased to about 11%, but the number of common induced genes to both genotypes was higher (17 transcripts). In the “stress response” category 8% and 14% of differentially expressed genes were observed in ‘PS’ and ‘Pat 81’ 1 DPI, respectively, with the majority down-regulated. At 3 DPI the percentage of genes categorized under ‘stress response’ was 8% in both genotypes, however the number of induced genes was twice the number of repressed genes in ‘Pat 81’. The number of genes classified in the “defence” group ranged from 2.7% to 3.8% and most of them were down-regulated. Transcripts categorized under “hormone metabolism” were mainly down-regulated in both genotypes and sampling dates, although some of them were up-regulated at 3 DPI in both genotypes. Genes belonging to these four categories are discussed in more detail below.

### Transcriptional evidence for Ca^2+^ and jasmonic acid in signalling pathogen responses

Calcium (Ca^2+^) plays a crucial role as a messenger in transduction during various developmental processes and in response to stress, including biotic stresses caused by fungi, bacteria and viruses [34,35]. In this study we identified seven transcripts up-regulated in response to *M. cannonballus* infection with similarity to Ca^2+^ sensor genes (Table 1). Three of them, coding for calmodulin (CaM)-like proteins (cCL1433Contig1, cCL417Contig1 and c46d_38-D02-M13R), had great transcript abundance exclusively in ‘Pat 81’ 1 DPI. At 3 DPI, two transcripts with homology to calcineurin B-like (CBL) protein (cCL3052Contig1) and CBL-interacting serine/threonine protein kinases (cCL3317 contig1) also had greater transcript abundance specifically in ‘Pat 81’ (Table 1), whereas the CBL-interacting serine/threonine protein kinase coded by cHS_34-D08-M13R was found to be up-regulated in both ‘Pat 81’ and ‘PS’. An Additional transcript coding for a calcium-dependent protein kinase (CDPK, cCL_57-B01-M13R) was found up-regulated in ‘PS’3 DPI. Recent evidence suggests that Ca^2+^ signalling *via* CDPKs, CBL/CBL-interacting protein kinases and CaM are involved in different aspects of biotic defence response [36,37]. Up-regulation of these genes mainly in the ‘Pat 81’ genotype suggests that Ca^2+^ signalling may play a part in the melon-*M. cannonballus* interaction.

**Table 1:** **List of selected melon transcripts modulated after *M. cannonballus *inoculation**

**ID**	**Melon**	**Annotation**	**Piel de sapo**	**Piel de sapo**	**Pat 81**	**Pat 81**
**MELOGEN**	**Unigene 4.0**		**1 DPI**	**3 DPI**	**1 DPI**	**3 DPI**
cCL1433Contig1	MU43495	Calmodulin-like protein 1	*	*	2.60	*
cCL417Contig1	MU47725	Probable calcium-binding protein CML27	*	*	2.09	*
c46d_38-D02-M13R	MU47725	Probable calcium-binding protein CML27	*	*	2.13	*
cCL3052Contig1	MU51164	Calcineurin B-like protein 7	*	*	*	2.20
cHS_34-D08-M13R	MU50771	CBL-interacting serine/threonine-protein kinase 1	*	2.26	*	3.71
cCL3317Contig1	MU51655	CBL-interacting serine/threonine-protein kinase 1	*	*	*	3.22
cCI_57-B01-M13R	MU59100	Calcium-dependent protein kinase 24	*	2.60	*	*
cA_12-H07-M13R	MU56451	Probable WRKY transcription factor 28	0.44	*	*	0.43
cCI_23-B11-M13R	MU58415	Probable WRKY transcription factor 51	*	*	*	0.09
cPSI_30-E06-M13R	MU65305	WRKY70 (*Citrullus lanatus*)	*	*	*	0.25
cCL935Contig2	MU44501	JAZ10 (JASMONATE-ZIM-DOMAIN PROTEIN 10)	*	*	0.43	0.26
cCL935Contig1	MU44501	JAZ10 (JASMONATE-ZIM-DOMAIN PROTEIN 10)	*	*	*	0.31
cPS_05-A06-M13R	MU64131	CYP82C2 (*Arabidopsis thaliana*)	*	3.27	*	8.57
cA_30-B04-M13R	MU56766	CYP82C2 (*Arabidopsis thaliana*)	*	2.08	*	3.51
cPS_23-C06-M13R	MU64454	Highly similar to Putative CCCH-type zinc finger transcription factor (*Gossypium hirsutum*)	*	*	*	3.61
cCL3498Contig1	MU50876	Highly similar to Syntaxin-121 (*Arabidopsis thaliana*)	*	*	2.29	*
cPS_18-B02-M13R	MU64359	MLO-like protein 4 (*Arabidopsis thaliana*)	*	*	*	0.49
cFR17N13	MU60754	MLO-like protein 6(*Arabidopsis thaliana*)	*	*	*	0.49
cCL4557Contig1	MU43859	Highly similar to Thaumatin-like protein (*Arabidopsis thaliana*)	*	*	*	2.27
cSSH1P11	MU65570	ATHSP22.0 (*Arabidopsis thaliana*)	2.44	*	2.68	9.37
cPSI_23-F06-M13R	MU45971	Heat shock cognate 70 kDa protein	*	5.55	*	8.33
cPSI_32-H04-M13R	MU45881	HSP18.2 (heat shock protein 18.2) (*Arabidopsis thaliana*)	*	18.55	*	10.69
cCL172Contig1	MU45179	HSP18.2 (heat shock protein 18.2) (*Arabidopsis thaliana*)	3.03	13.05	*	10.54
cCL3362Contig1	MU47164	5.7 kDa class I-related small heat shock protein-like (HSP15.7-CI) (*Arabidopsis thaliana*)	2.04	3.35	*	7.07
cCL5902Contig1	MU53662	ATHSP22.0 (*Arabidopsis thaliana*)	2.81	*	*	19.76
cCL3733Contig1	MU45345	Peroxidase 5 (*Vitis vinífera*)	0.048	0.011	*	0.06
cPSI_21-C11-M13R	MU54798	Peroxidase 64 (PER64) (*Arabidopsis thaliana*)	*	0.14	*	0.13
cPS_30-C04-M13R	MU46660	Netting associated peroxidase - *Cucumis melo*	0.46	0.27	0.38	0.49
cAI_08-H07-M13R	MU44823	Glutathione S-transferase GST 13 - *Glycine max*	0.27	*	0.36	0.23
cA_02-A09-M13R	MU43303	1-aminocyclopropane-1-carboxylate synthase CMA101 - * Cucurbita maxima*	0.14	*	0.09	*
cFR15J17	MU60665	1-aminocyclopropane-1-carboxylate oxidase 1 - *Cucumis melo*	0.15	*	0.26	*
cCL451Contig1	MU46283	1-aminocyclopropane-1-carboxylate oxidase 1 - *Cucumis melo*	0.29	*	0.26	0.29

* Not differentially expressed genes.

Note: The numbers indicate fold change of differentially expressed genes.

Members of the complex family of WRKY transcriptional factors play a broad and pivotal role in regulating defence [38]. Most of the WRKY-like transcripts identified in this work were found to have lower transcript abundance in infected samples. The transcript cPSI_30-E06-M13R, which was down-regulated in ‘Pat 81’ 3 DPI, showed high homology to *ClWRKY70* from *Citrullus lanatus* (Table 1), a salicylic acid (SA)-inducible gene that increases resistance to pathogens by overexpression in *Arabidopsis*[39]. *WRKY70* from *Arabidopsis* represses jasmonic acid (JA)-responsive genes and is itself down-regulated by JA, acting at a convergence point determining the balance between SA- and JA-dependent defence pathways. *WRKY70* is also required for R gene-mediated resistance [40-42]. Down-regulation of this transcript in ‘Pat 81’ points to the prevalence of JA signalling cascade over SA pathway for activating the early defence mechanisms triggered by *M. cannonballus* infection. In general, SA-dependent gene expression responses are effective against biotrophic pathogens, whereas defence genes activated by JA are effective against necrotrophic pathogens. Some exceptions to this statement have been documented, and frequently pathogens cannot be merely classified as biotrophic or necrotrophic as in the case of *M. cannonballus*[43,44].

Additional transcriptional changes indicate a role of JA pathways in the response to *M. cannonballus*. For example, among genes specifically down-regulated in ‘Pat 81’ 1 and 3 DPI, transcripts cCL935Contig1 and cCL935Contig2 had homology to *JASMONATE-ZIM DOMAIN 10* (*JAZ10*) of *Arabidopsis* (Table 1). JAZ proteins are negative regulators of the JA signal cascade through the interaction with certain transcription factors such as MYC2. Fast down-regulation of these genes upon *M. cannonballus* infection suggests the possibility of proteasome-independent pathways for the activation of JA-mediated plant defence responses in ‘Pat 81’ genotype [45].

The transcripts cPS_05-A06-M13R and cA_30-B04-M13R, encoding two related cytochrome P450 proteins, were induced 2 to 4-fold in ‘PS’ and 3 to 9-fold in ‘Pat 81’ 3 DPI. These transcripts show a high similarity to *CYP82C2* from *Arabidopsis*, involved in JA-induced expression of defence genes and accumulation of indole glucosinolates. A mutation in *CYP82C2* gene reduces plant resistance to the necrotrophic fungus *Botrytis cinerea* and alters root growth sensitivity to exogenous JA, whereas *CYP82C2* overexpression improves resistance to *B. cinerea*[46]. These data suggest the preferential activation of the JA signalling pathway 3 days after *M. cannonballus* infection of melon roots, instead of SA-dependent cascades. Interestingly, the differential quantitative and qualitative expression of *CYP82C2*-like and *JAZ10*-like genes in ‘Pat 81’ and ‘PS’ genotypes suggest that JA response might be partially responsible for their observed differences in resistance, although sequence differences between the two genotypes affecting array hybridization may also account for part of this variation.

Different studies indicate that JA- and ethylene-signalling frequently operate synergistically to induce the effector genes of defence responses [47], however we found no transcriptional evidence under our experimental conditions: several transcripts (cA_02-A09-M13R, cFR15J17, cCL451Contig1) coding for ACC synthase (ACS) and ACC oxidase (ACO), the enzymes catalysing the last two steps in the ethylene biosynthetic pathway, were found repressed in ‘Pat 81’ and Piel de sapo’ at different infection times (Table 1). Other authors have found also different transcripts with similarity to ACC oxidase genes differentially expressed after infection of melon with *Fusarium oxysporum* f. sp. *melonis*[48]. However, this does not preclude post-transcriptional and translational processes altering the activity of these enzymes and the production of ethylene. Further work is required to elucidate the roles of these hormones during the melon-*M. cannonballus* interaction.

#### Defence responses show transcriptional similarity to previously identified pathogen responses

In ‘Pat 81’ a transcript (cCL3498Contig1) homologous to *AtSYP121*/*PEN1*, encoding the protein syntaxin 121 from *Arabidopsis*, was induced at early stages of infection (1 DPI), but did not change its expression significantly in the susceptible ‘PS’ (Table 1). *PEN1* was identified in a screening for *penetration* (*pen*) mutants, required for the resistance to fungal penetration in the non host interaction between *Arabidopsis* and *Blumeria graminis* f. sp. *hordei* (*Bgh*) [49]. PEN1 protein is a constituent of a SNARE complex that contributes to the formation of cell wall appositions [49,50]. The ortholog of *PEN1* in barley (*ROR2*) was described as required for basal penetration resistance against *Bgh* in *mlo* (*mildew resistance locus o*) mutants [49,51]. In addition to barley, loss-of-function mutations of *MLO* genes conferred broad-spectrum resistance to powdery mildew in *Arabidopsis*, tomato and pea (*Pisum sativum*) [52-56]. In our study, 2 transcripts (cPS_18-B02-M13R and cFR17N13) with similarity to *MLO* genes were found to be down-regulated in ‘Pat 81’ at 3 DPI. A Blastx comparison of these ESTs against the *Arabidopsis*, tomato and pea protein databases using an E-value cut-off of 10^-5^ found only components of the MLO gene family from these three species. The first *Arabidopsis* blastx hits of cPS_18-B02-M13R and cFR17N13 were respectively MLO4 and MLO6. *MLO4* was recently described to affect growth responses of the *Arabidopsis* root in response to mechanical stimuli [57]. While the disruption of *MLO6*, together with mutants in the related *MLO2* and *MLO12 genes*, was required for the resistance to powdery mildew [53].

To date, there are no evidences supporting the occurrence of cell wall appositions in melon roots infected with *M. cannonballus*; however it is tempting to speculate that an early expression of a *PEN1*-like could contribute to delay and prevent to some extent the penetration of the fungus in ‘Pat 81’, resulting in an altered development of the infection. The subsequent repression of *MLO*-like genes, detected two days later, could tune up this specific response in ‘Pat 81’. Recently, a *MLO*-like gene has been described in melon [58]. The expression of this gene, designated *CmMlo1*, was up-regulated under cadmium exposure, which suggested its participation in abiotic stress responses, but this transcript is different from the transcripts identified in this work.

A gene specifically expressed in ‘Pat 81’ (cPS_23-C06-M13R) had similarity to *GhZFP1*, encoding a CCCH-type zinc-finger transcription factor of *Gossypium hirsutum.* Recently, this protein was characterized as a relevant positive regulator conferring salt tolerance and fungal pathogen resistance to plants [59]. Overexpression of *GhZFP1* in transgenic tobacco enhanced tolerance to salt stress and resistance to *Rhizoctonia solani*. Two possible interactors of GhZFP1 protein: GZIRD21A, similar to responsive to dehydration protein 21A, and GZIPR5, a pathogenesis-related protein 5 (PR5)-like were also identified [59]. Interestingly, a transcript with high similarity to PR5 of *Arabidopsis* was found up-regulated in ‘Pat 81’.

Several significantly differentially expressed transcripts with high similarity to pathogenesis-related (PR) genes (glucanases and chitinases among others) were down-regulated in both genotypes except for a transcript with homology to *PR5* of *Arabidopsis* (cCL4557Contig1), coding for a thaumatin-like protein, which was up-regulated in ‘Pat 81’ at 3 DPI. Such reduction of PR-related transcripts suggests that PR-specific defence mechanisms are not activated within the first 3 days after *M. cannonballus* inoculation. The down-regulation of PR genes was also reported by Schlink [60] in infected roots of *Fagus sylvatica* by *Phytophthora citricola* (hemibiotrophic oomycete), where they hypothesized that down-regulation would alter the pathogens’ chance to escape recognition. Nevertheless, in our system, additional studies on the late pathogenic response are required to elucidate the role of specific PR proteins in melon-*M. cannonballus* interaction.

#### Non-pathogen related stress responses are consistent between genotypes

Several transcripts related to non-pathogen stress responses were differentially expressed after fungal infection. These included members of the small and large heat-shock protein families (HSP), which were up-regulated in both genotypes (cSSH1P11, cPSI_23-F06-M13R, cPSI_32-H04-M13R, cCL172Contig1, cCL3362Contig1, cCL5902Contig1). We also found transcripts coding for proteins related to the oxidative stress and the regulation of reactive oxygen species (ROS) as peroxidase-like and glutathione S-transferase (cCL3733Contig1, cPSI_21-C11-M13R, cPS_30-C04-M13R, cAI_08-H07-M13R) to be significantly down-regulated in both genotypes.

## Conclusions

The results show common and divergent responses of the susceptible and resistant melon genotypes to infection with *M. cannonballus*. Transcriptomic differences are more apparent at an early stage of infection. Transcriptional differences in the activation of the JA-mediated response in ‘Pat 81’ compared to ‘PS’ suggest that JA response may be partially responsible for their observed differences in resistance. Several transcripts, previously implicated in plant fungal resistance, were also significantly differentially expressed in ‘Pat 81’, also potentially resulting in an altered infection development. Further studies are needed to quantify differences in tissue hormone concentrations between the two genotypes, as implicated in the differential expression of JA regulated genes, and identify the functional roles of many of the transcripts observed to be expressed more abundantly in ‘Pat 81’ melon compared to the susceptible ‘PS’ genotype. Recently the genome sequence of melon has been reported [61]. The authors predicted 27,427 protein-coding genes. Thus, this work offers a partial view on the whole picture of the transcriptomic changes occurring in our experimental model. Nevertheless these data along with future functional studies could lead to the identification/characterization of defence genes involved in resistance of melon to *M. cannonballus* vine decline disease.

## Methods

### Plant material

Two melon accessions, *Cucumis melo spp. melo* ‘Piel de sapo cv piñonet’ (‘PS’), fully susceptible to the infection by *M. cannonballus* and *C. melo spp. agrestis* ‘Pat 81’, resistant to the infection by *M. cannonballus* were used in this study. These accessions are maintained by Cucurbits Breeding group at COMAV-UPV.

### *In vitro* inoculation of *C. melo* roots with *M. cannonballus*

In a preliminary study, seeds from ‘PS’ were surface-sterilized with 20% bleach and a drop of Tween-20 for a minute and after rinsing, the seeds were placed in Petri dishes with wet filter paper under sterile conditions. After 6 days, seedlings were inoculated by direct contact with *M. cannonballus* mycelium grown on PDA (potato dextrose agar). To ensure the correct inoculation each root was rolled in germination paper with 2 discs (1 cm^2^ aprox.) of PDA with active growing mycelium. Mock treatments were prepared in the same way using PDA without mycelium. Plants were placed in a container containing a half diluted nutrient solution composed of: 3 mM KNO_3_, 2 mM Ca(NO_3_)_2_.4H_2_O, 0.5 mM MgSO_4_.7H_2_O, 0.5 mM (NH_4_)H_2_PO_4_, 25 μM KCl, 12.5 μM H_3_BO_3_, 1 μM MnSO_4_.H_2_O, 1μM ZnSO_4_.7H_2_O, 0.25 μM CuSO_4_.5H_2_O, 1.3 μM (NH_4_)_6_ Mo_7_O_24_.4H_2_O and 25 μM Fe-NaEDTA. Axenic conditions were maintained. Plants were grown in a climatic chamber (28°C, 16/8 h light/dark). Infected roots of ‘PS’ melon (four plants per time-point) and their respective mock-inoculated controls (one plant per time-point) were collected at 1, 3 and 5 days post inoculation (DPI). Total DNA was extracted using the DNeasy Plant Mini Kit (Qiagen, Hilden, Germany) and the presence of the fungus and the infection levels were assessed by quantitative PCR with specific *M. cannonballus* primers as described previously [31].

### Sample collection and RNA isolation

Root samples for the extraction of total RNA used to hybridize to the melon microarray were taken from plants grown using the inoculation method described above. For each biological sample we collected roots from 4 to 6 plants per genotype (‘PS’ and ‘Pat 81’), treatment (inoculated and mock-inoculated as control) and time post inoculation (1DPI and 3DPI). Three biological replicates were used for each genotype/treatment/time combination and independently hybridized to the melon microarray. Total RNA was isolated using TRI Reagent (Sigma-Aldrich Corporation, St. Louis, MO, USA) according to manufacturer’s protocols and further purified using RNeasy Mini Kit (Qiagen). RNA integrity and quality was checked on agarose electrophoresis. Quantity and purity of total RNA were determined by Nano-Drop ND-1000 spectrophotometer (NanoDrop Technologies, Wilmington, DE, USA). Total RNA samples were sent to Roche NimbleGen Systems where cDNA synthesis, Cy3 labelling and hybridizations were performed following the manufacturer’s procedures.

### Microarray data analysis

The melon microarray is an oligo-based (60-mer) microarray representing a total of 17,443 unigenes derived from 33,418 high-quality melon ESTs [29]. Sequences of these unigenes are listed in Additional file 7. Hybridization signal intensity was calculated using a GenePix 4000B (Molecular Devices, Sunnyvale, CA, USA) and the data were extracted using NimbleScan software (Roche NimbleGen). The intensity values obtained from the array scanning were normalized using the robust multiarray average (RMA) [62]. Normalized probe set data, provided by Roche NimbleGen Systems in RMA calls files, were imported into ArrayStar software 3.0 version (Dnastar, Madison, WI, USA), where statistical analysis was performed. Data from infected samples were normalized to their respective controls. Data were log2 transformed, thus normalized values are the log2 of the ratio between infected and control samples at a given time-point. Significantly differentially expressed genes were identified using an unpaired t-test with a Benjamini-Hochberg multiple testing corrected p-value cut-off of 0.05 [63] and a fold change cut-off of 2. The microarray data were deposited at ArrayExpress (http://www.ebi.ac.uk/microarray-as/ae/) under the accession number E-MEXP-3732. Transcripts differentially expressed were annotated based on the MELOGEN database [27], and genes discussed in detail were re-annotated using Cucurbit Genomic Database Melon Unigene v. 4.0 [64]. Additionally, we performed a functional classification of transcripts following the Gene Ontology (GO) scheme with Blast2GO package [33]. This information and previously published data allowed us to classify manually the genes in functional groups.

Principal component analysis (PCA) of all samples was generated using TMeV 4.0 software from TIGR [65]. The Venn diagrams were made manually using the output lists of the statistical analysis.

### Quantitative RT-PCR

To validate the microarray experiments, the transcript levels of nine selected genes were quantified using qRT-PCR. First strand cDNA was synthesized from 1 μg of total RNA with the Oligo (dT)_20_ (50 μM) primer using the Expand Reverse Transcriptase (Roche Applied Science, Penzberg, Germany), according to the manufacturer’s instructions. Quantitative PCR was performed with an ABI PRISM 7000 Sequence Detector System (Applied Biosystems, Foster City, CA, U.S.A), using FastStart Universal SYBR Green Master (ROX) (Roche Applied Science) and 2 μl of diluted 1:10 cDNA for each PCR reaction. The relative expression level was determined using the *cyclophilin* (cCL1375) housekeeping gene from melon as reference [27]. The gene specific primers for PCR amplification were designed using Primer3 v.0.4.0 [66] (Additional file 6). The fold changes in each infected sample compared to the expression level detected in the corresponding sample under control conditions were calculated using the 2^-ΔΔCT^ method [67]. Intra-assay variation was evaluated by performing all amplification reactions in duplicate.

## Competing interest

The authors have declared that no competing interest exists.

## Authors’ contributions

CR, BP, AF and FN were involved in the conception and design of the study. CR and AF were responsible for growing melon plants and performed melon infection. CR prepared RNA extraction and qRT-PCR. CR and JH performed microarray statistical analysis. GR performed functional classification of transcripts and revised the manuscript. CR was responsible for drafting and revising the manuscript with contributions from co-authors. All read and approved the final manuscript.

## Supplementary Material

Additional file 1 **Table S1.** List of deregulated genes in 'PS' at 1 DPI. Click here for file

Additional file 2 **Table S2.** List of deregulated genes in 'Pat 81' at 1 DPI. Click here for file

Additional file 3 **Table S3.** List of deregulated genes in 'PS' at 3 DPI. Click here for file

Additional file 4 **Table S4.** List of deregulated genes in 'Pat 81' at 3 DPI. Click here for file

Additional file 5 **Table S5.** List of differentially expressed genes in common between this work and [29]. Click here for file

Additional file 6 **Table S6.** Genes and primers used for qRT-PCR. Click here for file

Additional file 7 **Table S7.** Sequence of unigenes represented in the microarray. Click here for file
